# Germs in a Baby's Jaw

**Published:** 1890-08

**Authors:** 


### Editorial, Etc.

GERMS IN A BABY'S JAW.—HOW THE FIFTY-TWO TEETH COME OUT INTO THE DAYLIGHT.—"The development of the teeth germs, from infancy to mature life, is one of the most interesting phases of human growth. Pass the finger along the tiny jaw of the newcomer. Not only is there nothing which presages future teeth, says an exchange, but the jaws themselves seem too delicate and frail to become the sockets for such hard working portions of the anatomy. Yet there are fifty-two tooth germs hidden there, waiting their time, though not all of them could be detected by the most skillful detection. Twenty of these are for the temporary teeth, with which, in due time, the child will begin to gnaw or chew his way through life ; the others include the permanent set and the molars, none of which begin to make their presence known till the child is about six years old, and the "wisdom" teeth do not usually appear till about eighteen.

The little pulp-germ grows and develops till it approximates the shape of the tooth it is to become; then it begins to calcify, forming the dentine part of the crown, while the enamel is deposited by an independent process. The surface of the crown attains its full size before the process of elongation commences. Then gradually it pushes its way outward through the gum, absorbing the tissue as it advances till the pure white enamel peeps out, to the mothers great delight. The first sight of the incisors usually appears at about the age of five months, and the temporary set of twenty is completed at about three years of age, but there is no uniformity or certainty in regard to the age or which of the four central incisors will first appear. The process of "teething" is almost invariably one of disturbance to the health of the child; he is liable to fret and worry, especially if the outer membrane or skin of the gum proves tenacious and does not readily absorb. In this case it should be lanced—an operation which is humane, in that it relieves the discomfort of the child, and is entirely harmless, as there is seldom any hemorrhage worth the name, and if there should be a slight flow of blood, it readily yields to simple treatment. The application of a dust of powdered alum is usually sufficient."

